# Feasibility of a Comprehensive Targeted Cholera Intervention in The Kathmandu Valley, Nepal

**DOI:** 10.4269/ajtmh.18-0863

**Published:** 2019-03-18

**Authors:** Mellisa Roskosky, Bhim Acharya, Geeta Shakya, Kshitij Karki, Kazutaka Sekine, Deepak Bajracharya, Lorenz von Seidlein, Isabelle Devaux, Anna Lena Lopez, Jacqueline Deen, David A. Sack

**Affiliations:** 1 Department of International Health, Johns Hopkins Bloomberg School of Public Health, Baltimore, Maryland;; 2 Epidemiology Disease Control Division, Department of Health Services, Ministry of Health, Kathmandu, Nepal;; 3 National Public Health Laboratory, Department of Health Services, Ministry of Health, Kathmandu, Nepal;; 4 Group for Technical Assistance, Kathmandu, Nepal;; 5 United Nations International Children’s Emergency Fund (UNICEF) Sierra Leone, Freetown, Sierra Leone;; 6 Mahidol Oxford Tropical Medicine Research Unit, Faculty of Tropical Medicine, Mahidol University, Bangkok, Thailand;; 7 Nuffield Department of Medicine, Centre for Tropical Medicine and Global Health, University of Oxford, Oxford, United Kingdom;; 8 Institute of Child Health and Human Development, National Institutes of Health, University of the Philippines, Manila, Philippines

## Abstract

A comprehensive targeted intervention (CTI) was designed and deployed in the neighborhoods of cholera cases in the Kathmandu Valley with the intent of reducing rates among the neighbors of the case. This was a feasibility study to determine whether clinical centers, laboratories, and field teams were able to mount a rapid, community-based response to a case within 2 days of hospital admission. Daily line listings were requested from 15 participating hospitals during the monsoon season, and a single case initiated the CTI. A standard case definition was used: acute watery diarrhea, with or without vomiting, in a patient aged 1 year or older. Rapid diagnostic tests and bacterial culture were used for confirmation. The strategy included household investigation of cases; water testing; water, sanitation, and hygiene (WASH) intervention; and health education. A CTI coverage survey was conducted 8 months postintervention. From June to December of 2016, 169 cases of *Vibrio cholerae* O1 were confirmed by bacterial culture. Average time to culture result was 3 days. On average, the CTI Rapid Response Team (RRT) was able to visit households 1.7 days after the culture result was received from the hospital (3.9 days from hospital admission). Coverage of WASH and health behavior messaging campaigns were 30.2% in the target areas. Recipients of the intervention were more likely to have knowledge of cholera symptoms, treatment, and prevention than non-recipients. Although the RRT were able to investigate cases at the household within 2 days of a positive culture result, the study identified several constraints that limited a truly rapid response.

## INTRODUCTION

Cholera, caused by ingestion of the bacterium *Vibrio cholerae*, presents clinically as the rapid onset of acute watery diarrhea (AWD) and vomiting. Without treatment, the disease can progress rapidly and lead to death from severe dehydration in as little as 4 hours from the onset of symptoms.^1^ During an outbreak in Guinea-Bissau, researchers found that cholera cases tended to cluster spatially with at least 30% of households in each cluster having at least one case.^2^ A later study in Matlab, Bangladesh, (a rural area) showed that those living closest to a case (within 50 meters) had 36-fold higher chance of becoming infected with cholera than those living in other areas of the community.^3^ This risk was highest during the first 3 days after the index case was identified. Similar results were seen in urban Kolkata, India, where an increased risk for cholera was seen within 25–50 m of a case and persisted for a month, and up to 200 m for 5 days in Chad and D.R. Congo.^4–6^ These observations highlight that rapid detection of cholera cases and rapid response is needed to control the spread of disease.

Cholera is endemic in Nepal, with a model-estimated 30,000 cases and 911 deaths per year during the monsoon months of May through September.^7^ Although the disease occurs nearly every year in the Kathmandu Valley and is detected sporadically in other parts of the country, exactly where it will occur is unpredictable.

Despite much progress, the global supply of oral cholera vaccine (OCV) remains limited as compared with the global at-risk population.^8^ By designing an intervention that focuses on halting an outbreak early rather than preventing one entirely, it may be possible to limit the transmission from index cases and reduce the overall rates of disease by targeting the high-risk groups who live near a case. A reactive vaccination strategy, when partnered with other important cholera prevention measures, forms the backbone of a comprehensive targeted intervention (CTI) approach to cholera control and has the potential to halt the spread of cases if deployed rapidly. Founded on a strengthened surveillance system, the CTI approach combines pointed health behavior messaging with traditional water, sanitation, and hygiene (WASH) interventions and a single-dose OCV campaign (estimated to have a short term effectiveness of 87%) to prevent the spread of cholera once it strikes.^9^ Delivery of a single dose of OCV to case households and neighbors after a case is detected has been previously been shown to be feasible in an urban setting in South Sudan.^10^ A single dose of OCV using a reactive ring strategy was included in the CTI plan, but the vaccine could not be obtained in time for the summer 2016 outbreak. Although preventing an outbreak by vaccinating a defined “hotspot” is preferable, this strategy is only successful when outbreaks occur consistently in an area. Outbreaks tend to occur sporadically during the monsoon season in varying areas of the city; thus, a preventive hotspot campaign for a large urban area such as Kathmandu is not feasible. By designing an intervention that focuses on halting an outbreak early, it may be possible to reduce transmission and lower morbidity and mortality significantly in Nepal and other countries in high-risk situations.

### Study objective and rationale.

Following a major earthquake, the Epidemiology and Disease Control Division (EDCD) of the Department of Health Services in Nepal adopted a CTI approach to cholera control for the 2016 monsoon season, expanding the role of the country’s existing rapid response team (RRT) network. This study was designed to assess the feasibility of that response. We hypothesized that a CTI strategy for controlling cholera in the Kathmandu Valley would facilitate a cooperative and unifying method for cholera control and could potentially reduce transmission in this area. Deploying an early warning system and RRT for cholera in post-earthquake Nepal faced many challenges—poor disease surveillance, limited laboratory capacity, and loss of health infrastructure to name a few.^11^ This study was designed as a feasibility study to determine whether clinical centers, laboratories, and field teams were able to facilitate rapid, multi-sectoral response within 2 days of a case being admitted to the hospital.

## METHODS

### Intervention summary.

Enhanced hospital-based surveillance for cholera cases took place at 15 sites throughout the Kathmandu Valley, consisting of Kathmandu, Lalitpur, and Bhaktapur districts. Focal persons (physicians and/or medical recorders) were identified at each hospital to be responsible for reporting suspected cases of cholera to the EDCD RRT. Physicians at the sentinel sites identified patients suspected of having cholera using a standard case definition: AWD, with or without vomiting, in a patient aged 1 year or older. Daily line listings of AWD cases were requested from each hospital, including zero-reporting, and a single suspected case of cholera triggered the CTI cascade. When such a patient was identified, a laboratory technician at the hospital was expected to perform both a rapid diagnostic test (RDT) and culture for cholera, in addition to sending a stool specimen to the National Public Health Laboratory (NPHL), for culture confirmation and serotyping. Within the same day (approximately 6 hours), any positive RDT result was reported to the EDCD by the appointed hospital focal person. Once notified, the RRT was expected to travel to the neighborhood of the index case the same day (or no more than 2 days) to initiate the CTI intervention. As this response was taking place at a time when the country was still recovering from the 2015 earthquakes, the country was still especially vulnerable to disease outbreaks. To limit the burden on the existing RRTs, an additional team was hired to assist specifically with the cholera response efforts. This six-person team consisted of microbiologists, health scientists, a geographic information system (GIS) specialist, and a data manager.

**Figure 1.**
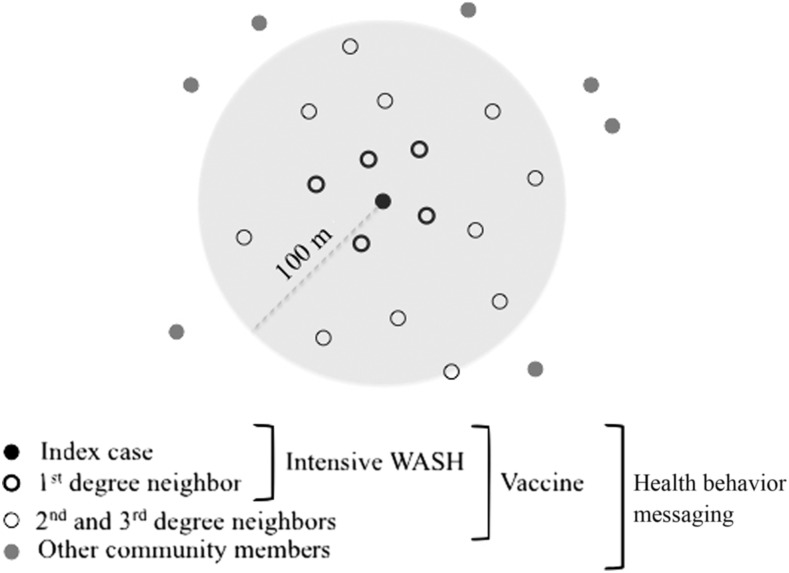
Proposed comprehensive targeted intervention ring strategy. A 100-m ring is approximately identified around an index case (shaded area). Intervention households are indicated by points, and specific interventions vary by distance from the index household (black point).

**Figure 2.**
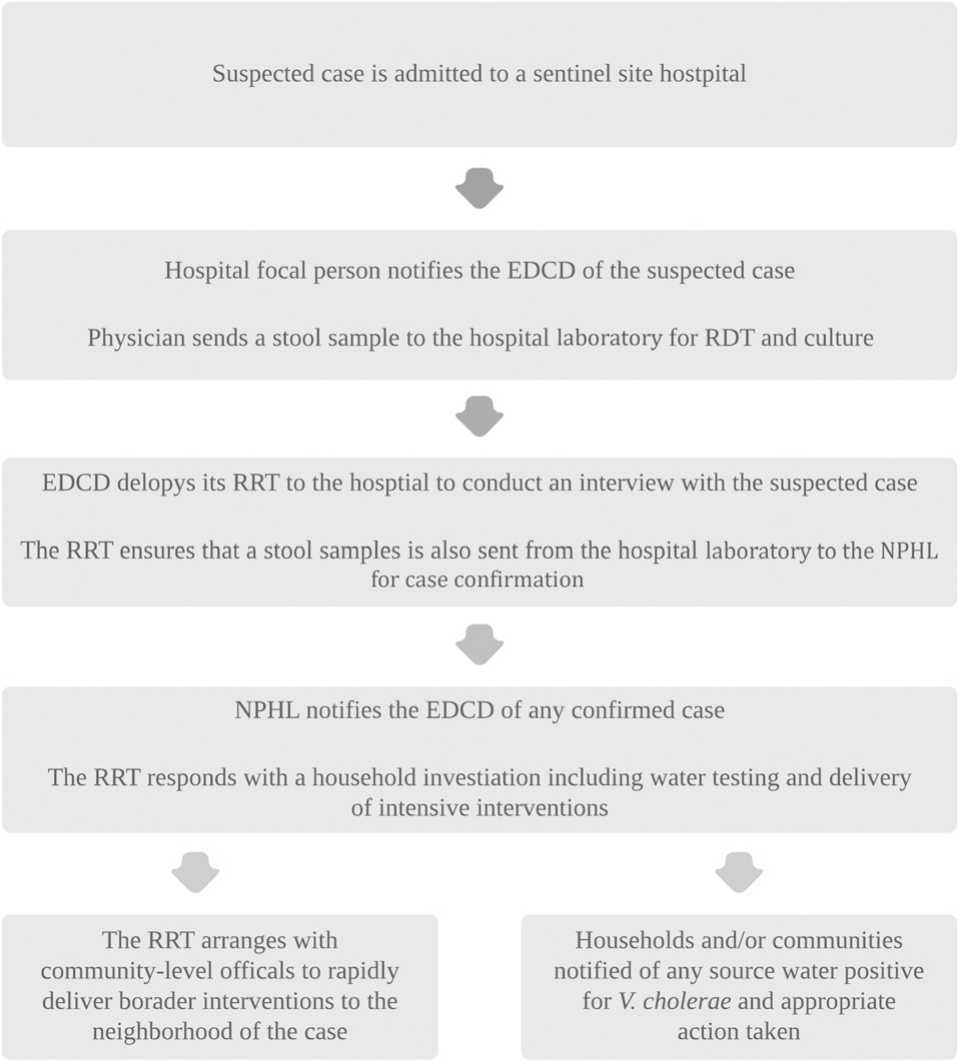
Flow of information in the proposed comprehensive targeted intervention ring strategy.

The CTI intervention included (Figures 1 and 2):

1. Hospital and household investigations of each case
2. An intensive WASH intervention to the case household and the first-degree neighbors
3. Community-level WASH activities and health education messaging in the neighborhood surrounding the case to encourage safe water, safe food, and hand-washing
4. Water testing for *V. cholerae* at each index household

### Case investigation.

Patients meeting the case definition for cholera were expected to have their clinical information recorded at the hospital by the RRT in what was termed the hospital investigation. Data collected here focus solely on the index case and included a summary of demographic characteristics, signs and symptoms, and approximate address. These data were aggregated and used to generate daily situation reports for the ministry and other relevant stakeholders.

The RRT was then to be deployed to the home of the index case within 2 days to conduct the household investigation. GIS locations of the homes were recorded to map the geographic distribution of the outbreak. If a family did not consent to a home visit, or if the RRT was unable to contact the family, the approximate location of the household was mapped based on the patients’ neighborhood. Household investigation data included information on water sources, water treatment, history of diarrhea within the household over the last 2 weeks, history of food consumption and travel, and sanitation and hygiene behaviors of the entire household.

### Water, sanitation, and hygiene intervention.

The family of the index case and their immediate neighbors (Figure 1) were then to be targeted for a door-to-door awareness campaign aimed at educating the high-risk groups on the risk of cholera transmission and methods for prevention. The names and addresses of patients were never identified, but the messaging indicated that cholera had been recently detected in the neighborhood. The RRT was expected to visit the households within 2 days of the initial index case in that area. Households were to receive orientation and equipment for point-of-use water treatment. This included chlorine tablets, basic buckets for water storage and hand washing, and soap. They were also expected to receive education, both verbal communication and a flyer for future reference, on hand washing at critical times, water treatment, food hygiene, and personal hygiene and sanitation.

### Health education intervention.

Female community health volunteers (FCHVs) and volunteers from local non-governmental organizations (NGOs) were trained by members of the cholera-specific RRT to deliver messages to the broader community surrounding the index case on various cholera prevention strategies. These messages included boiling, filtering, or treating water with chlorine, basic hygienic food preparation, parasite prevention methods, and the importance of vitamin A supplementation. Messages were to be delivered in a variety of ways to maximize coverage. Booths were set up in community areas with high foot traffic and the volunteers handed out flyers and answered questions. Awareness rallies were held with banners displaying prevention methods. Schools and food vendors were also targeted if they were within the target area. Presentations were given at meetings of community groups and schools, and radio messaging was projected from a vehicle (miking).

### Water sampling.

Surface water samples (3 L each) were collected from primary and secondary drinking water sources for the index case households, filtered through sterile gauze, and incubated in alkaline peptone water for 24 hours.^12^ The NPHL then performed culture analysis to preserve any *V. cholerae* isolates on thiosulfate citrate bile salt sucrose agar. Rapid response teams and FCHVs used a coliform presence/absence (H_2_S) test kit (Environment and Public Health Organization, Kathmandu, Nepal) as a visual demonstration of water unfit for drinking.^13^ These test kits were used at households and in the community on all water sources. The NPHL also performed a quantitative test for coliforms, the Idexx colilert-18 test.^14^

Total chorine tests were performed on household water sources and for tankers supplying water to the community.^15^ Results of testing at the community level were shared with that community and solutions to any water quality issues identified. If *V. cholerae* was found in any water source, families and the community were notified. Interventions for positive sources were dependent on the nature of the water source and were handled on a case by case basis.

### Follow up.

As part of the post-CTI program monitoring and evaluation, a survey was conducted in each of the areas targeted for a community-level WASH intervention. A field team visited each of the intervention areas and administered a simple questionnaire to households using a multistage cluster sampling method. A total of 400 households were targeted for the survey. First-stage clusters were the targeted wards, the lowest administrative unit used for planning and governance in Nepal, and the number of households sampled was proportionate to population size. For first-stage clusters larger than 20 households, an additional cluster was added within that ward, resulting in 30 total clusters. Second-stage clusters were chosen according to the WHO vaccine coverage survey guidelines.^16^ The survey asked residents whether their home was visited by a volunteer, whether they heard the health messaging in their neighborhood, whether they received specific information on health promotion from the volunteers, and whether they received water purification materials. It also included an assessment of their ability to answer basic hygiene promotion, water purification, and cholera prevention questions. Residents were consented to participate and had the option to refuse to answer a question if they desired.

### Analysis.

Detailed records on timeliness of the intervention were kept electronically by specific members of the RRTs for each phase of the response, and these were reviewed with the goal of answering questions regarding time to response. The number of cases over the course of the outbreak was graphed and cases were mapped by location of household using ArcGIS (ESRI, Redlands, CA). Basic demographic characteristics of AWD cases, cholera cases, and survey respondents were described. Feasibility was measured through indicators of timeliness and quality of implementation (Table 1). Survey data were summarized as a coverage rate which served as a proxy measure of quality. The effect of receiving the WASH intervention on cholera knowledge was assessed using generalized estimating equations with the logit link function and independent within-ward correlation matrices to account for clustering at the ward level. The dependent variables were they key knowledge indicators (yes or no) and the explanatory variable was the receipt of a WASH intervention. Both bivariate and multivariable models were used. Variables such as age, gender, years of education, and monthly household expenditure were used in the multivariable model. The estimates from the models were exponentiated to obtain odds ratios (ORs). Statistical analyses were performed using R version 3.5.1 (R Foundation for Statistical Computing, Vienna, Austria) and STATA version 15 (StataCorp LLC, College Station, TX).

**Table 1:** Comprehensive targeted intervention feasibility indicators

Indicators	Definition
Time from patient admission to case confirmation	Days (mean and range) from admission to case confirmation
The percentage of index households found and interventions implemented	Numerator: number of index households found
	Denominator: total number of cholera cases from the project area detected by the hospital labs
Time from case confirmation to household investigation	Days (mean and range) from case confirmation to household visit
The percentage of households in the target areas receiving WASH intervention in less than 48 hours after detection of the index case	Numerator: number receiving WASH in under 48 hours
	Denominator: total number receiving WASH
The percentage of households who report having heard WASH messaging at the household or community level	Numerator: number of households who received messaging
	Denominator: total number of households approached
The percentage of rings vaccinated in less than 3 days after detection of the index case	Numerator: number of rings vaccinated in less than 3 days
	Denominator: total number of rings vaccinated
Number of doses delivered per day during an oral cholera vaccine campaign	Doses (mean and range) delivered each day
The percentage of eligible household members of the index cases who received the single dose of vaccine	Numerator: number of eligible household members of the index cases who received the dose of vaccine
	Denominator: total number of household members of the cases
The percentage of eligible neighbors in the defined ring around the index cases who received the single dose of vaccine	Numerator: number of eligible neighbors in the defined ring around the index cases who received the dose of vaccine
	Denominator: total number of eligible neighbors in the defined ring around the index cases

WASH = water, sanitation, and hygiene.

### Ethical approval.

Institutional Review Board (IRB) approval was obtained from the Johns Hopkins University IRB and the Nepal Health Research Counsel for the use of de-identified data collected during the campaign.

## RESULTS

**Figure 3.**
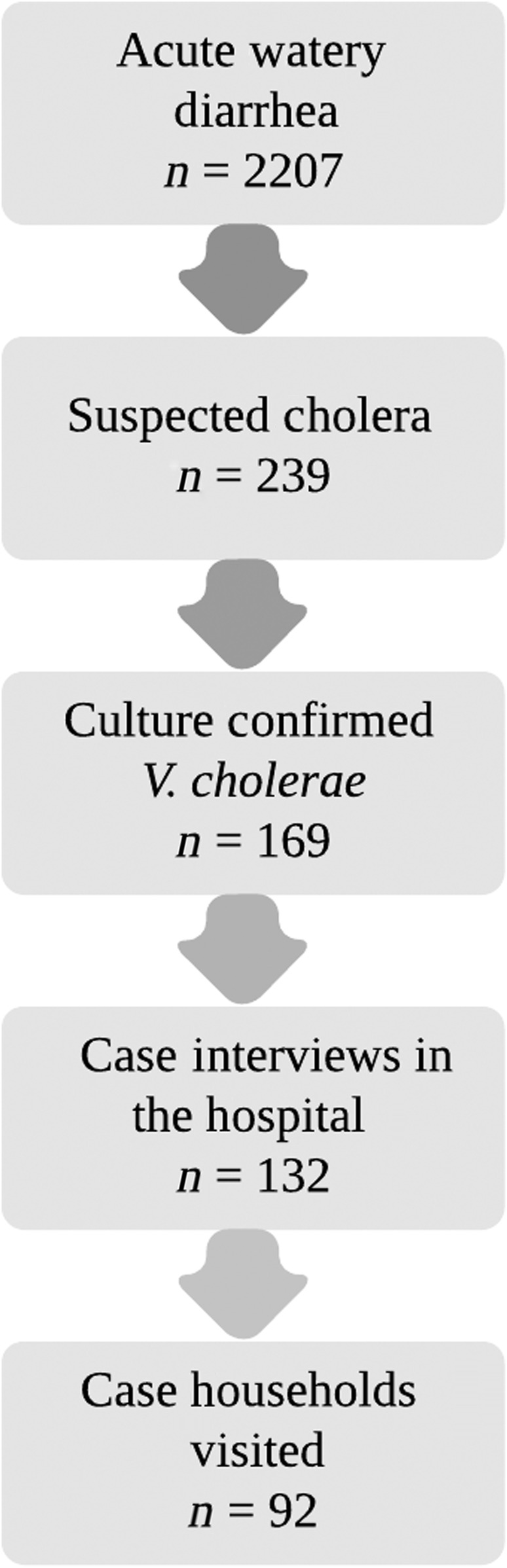
Flow of cases from hospital admission to household investigation.

**Figure 4.**
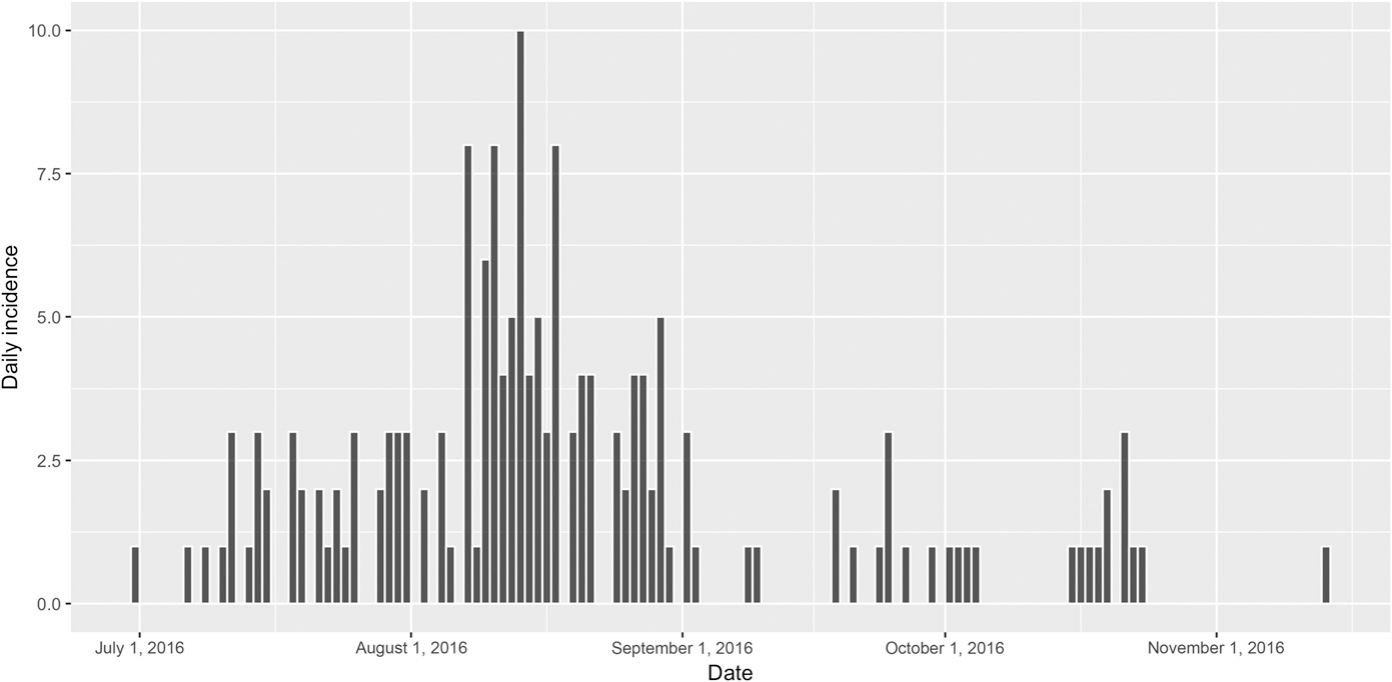
Epidemic curve in the Kathmandu Valley, Nepal, 2016. Confirmed cholera cases shown in bars, defined as all individuals who are positive for *Vibrio cholerae* by culture (*n* = 169).

**Figure 5.**
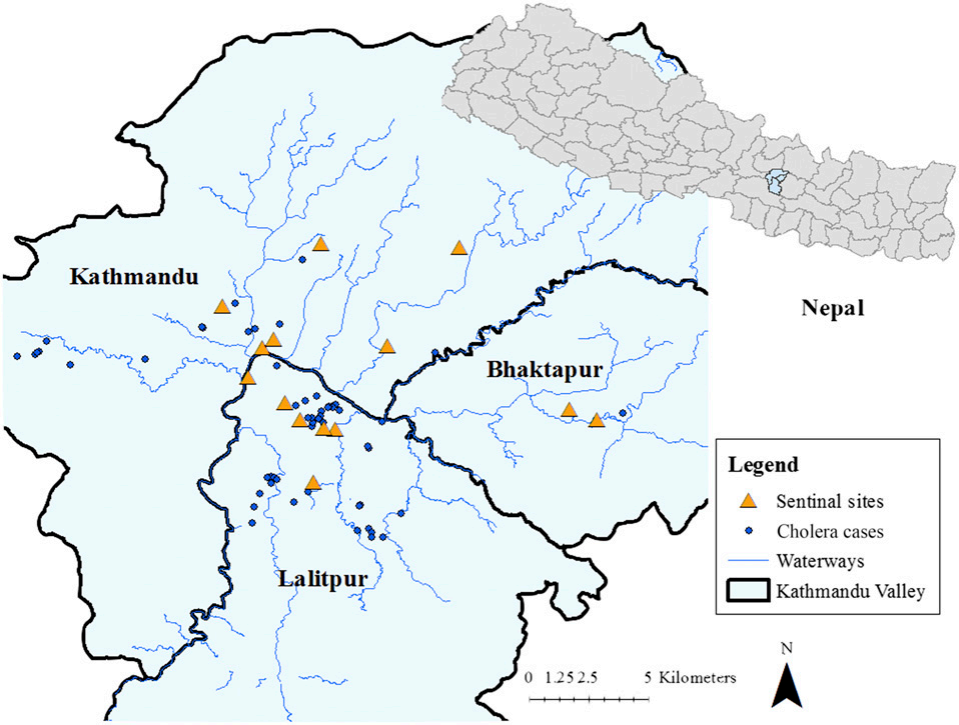
Geographic distribution of cholera cases in the Kathmandu Valley, 2016. Points indicate the location of the case. Triangles show the location of hospital sentinel surveillance sites. This figure appears in color at www.ajtmh.org.

A total of 2,207 cases of AWD were reported from the sentinel sites to the EDCD between June and November 2016 (Table 2, Figure 3). Of those AWD cases, 239 were classified as suspected cholera on the basis of clinical symptoms, namely dehydration status and the presence of rice-water stool. In total, 169 cases were culture confirmed as *V. cholerae* O1 Ogawa (Table 2, Figure 4). Rapid diagnostic test results were compared with bacterial culture for suspected cases with results for both tests (*N* = 194) and resulted in a sensitivity and specificity of 90% and 70%, respectively. Male–female ratio was similar between cholera and non-cholera diarrhea patients, but cholera patients were younger on average. Mostcases were detected from the Kathmandu Valley. The geographic distribution of cholera cases can be seen in Figure 5. More than 70% of cases were reported from Lalitpur district (120/169).

**Table 2:** Population characteristics

	Patient population		Survey respondents
	AWD*	Cholera	
N	2,207	169	394
Mean Age (SD)	35.20 (21.03)	25. 46 (14.03)	38.5 (13.51)
Gender			
Male	975 (44.5%)	79 (46.7%)	151 (38.3%)
Female	1,218 (55.5%)	90 (53.3%)	243 (61.7%)
Mean years of education (SD)	–	–	9.00 (3.48)
Mean monthly household expenditure† (SD)	–	–	27,789 (21,445)

* Acute watery diarrhea (AWD); including cholera.

† Nepali Rupees.

**Figure 6.**
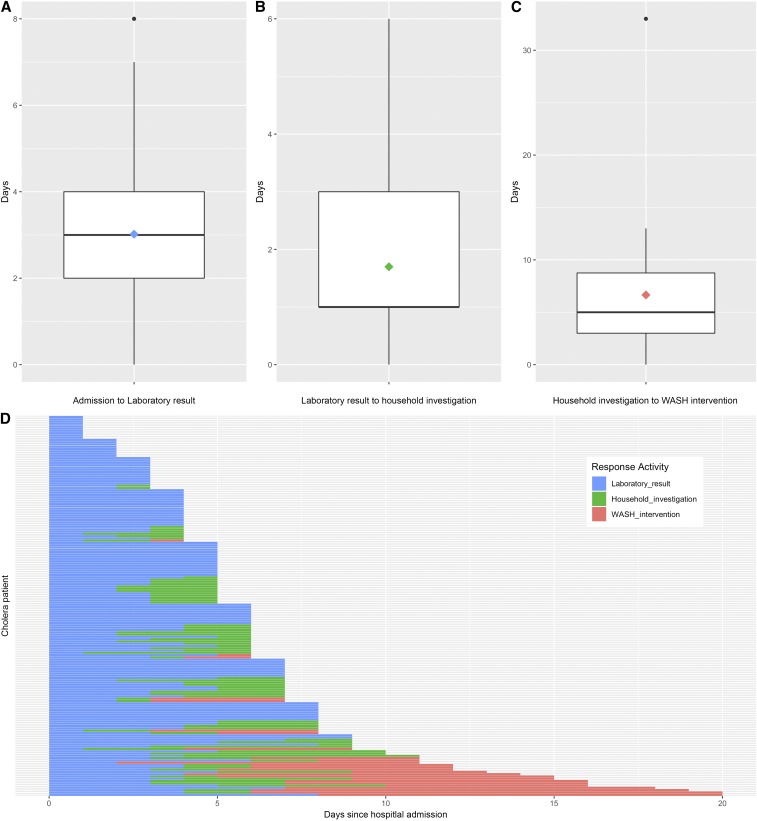
Comprehensive targeted intervention surveillance and response performance. (**A**–**C**) Box plots of each segment of the response activities, mean response time is represented by a colored diamond. (**D**) Each bar represents a study participant. The bars indicate the time from hospital admission of the index case to initiation of a water, sanitation, and hygiene (WASH) intervention in the neighborhood of that case. Colors correspond to individual segments of time and apply across plots as follows: time from hospital admission to result of laboratory culture (blue), time from laboratory result to household investigation, and time from household investigation to WASH intervention. Data are only shown for cases in which complete date information is available for at least one segment of the response activities (*n* = 165).

An evaluation of the speed with which the CTI was implemented can be seen in Figure 6. The average time from hospital admission to culture result was 3.0 days (SD: 1.9; range: 1–8 days). Rapid response teams interviewed a total of 132 confirmed cases in the hospital (78% of total cases), and of those cases, 92 household investigations were performed (54% of total cases) (Figure 3). On average, the RRT was able to visit the household 1.7 days after the culture result was received from the hospital (SD: 1.4; range: 0–6 days). It took an average of 3.9 days from hospital admission to household investigation (SD: 2.0; range: 1–9).

Water samples were collected from all case households that were investigated and 90.7% (117/129) of those household water samples were found unsuitable for drinking based on coliform count (more than one coliform per 100 mL). Three household water sources were positive for *V. cholerae* O1 Ogawa. Only 8.5% of drinking water samples tested had detectable levels of chlorine (10/118).

Water, sanitation, and hygiene and health behavior messaging campaigns were conducted in 18 areas of Lalitpur and Kathmandu districts. On average, these campaigns happened 9.0 days after the initial case in that area was admitted to the hospital (SD: 6.8; range: 0–37 days). A total of 394 households were surveyed (Table 2), of which 119 reported hearing WASH messaging during the monsoon season for a coverage rate of 30.2%. When asked which messaging was heard, survey respondents were most likely to report hearing about the importance of water purification and most often via miking or a household visit (Table 3). The results of the knowledge assessment can be seen in Table 4. The multivariable model after adjusting for the covariates showed that those who had received the WASH messaging were more likely to have heard of cholera, be able to name a treatment facility, and to report practicing at least one prevention method at home than those who did not receive an intervention. Those who recalled receiving an intervention were also more likely to correctly identify the high-risk season for cholera as the summer monsoon months. However, near perfect correlation between education and ability to identify the cholera season in the WASH intervention group led to a very high OR in the multivariable model compared with that in the bivariate model.

**Table 3:** Water, sanitation, and hygiene intervention coverage and messaging

Intervention	*N*	%
Household visit by female community health volunteers		
Respondents who received a visit	65	16.5%
Reported messaging during visit		
Hand washing	43	66.2%
Water purification	52	80.0%
Food hygiene	23	35.4%
Personal hygiene	31	47.7%
Sanitation	19	29.2%
Cholera education	8	12.3%
Reported supplies provided during visit		
Chlorine tablets	41	63.1%
Water storage bucket	1	1.5%
Soap	4	6.2%
Miking		
Respondents who heard miking	72	18.3%
Reported messaging heard		
Hand washing	43	59.7%
Water purification	67	93.1%
Food hygiene	23	31.9%
Cholera education	20	27.8%
Parasite prevention	2	2.8%
Vitamin A supplementation	1	1.4%
Other water, sanitation, and hygiene Interventions		
Booth campaign	15	3.8%
Awareness rally	13	3.3%
Community group meeting	16	4.1%
School intervention	6	1.5%

*N* = 394 total respondents.

**Table 4:** Knowledge of cholera symptoms, causes, prevention and treatment

	No intervention (*N* = 275)		Received any water, sanitation, and hygiene intervention (*N* = 119)		Crude OR	Adjusted OR*
	*N*	%	*N*	%		
Heard of cholera	224/275	81.5%	109/119	91.6%	2.48†	2.38†
Among those who had heard of cholera						
Could identify cholera season	203/224	90.6%	106/109	97.2%	3.66†	16.3†
≥ 1 correct symptom named‡	193/224	86.2%	93/109	85.3%	0.93	0.90
≥ 1 correct cause named§	207/224	92.4%	104/109	95.4%	1.71	3.99
≥ 1 correct treatment method named^‖^	63/224	28.1%	28/109	25.7%	0.88	0.89
≥ 1 correct treatment facility named¶	120/224	53.6%	84/109	77.1%	2.88†	3.47†
≥ 1 correct prevention method named#	209/224	93.3%	104/109	95.4%	1.49	4.07
Reported practicing ≥ 1 prevention method at home**	231/275	84.0%	113/119	95.0%	3.59†	3.63†

* Odds ratios (ORs) are adjusted for age, gender, education, and monthly household expenditure.

† Significant at alpha = 0.05.

‡ Diarrhea, vomiting, dehydration, and rice-water stool.

§ Contaminated water, contaminated food, and poor handwashing practices.

^‖^ Oral rehydration solution and intravenous fluids.

¶ Government hospital, private hospital, health post, and cholera treatment center.

# Drinking safe water, cooking food thoroughly, hand-washing, sanitary latrines, and vaccination with oral cholera vaccine.

** Boiling drinking water, treating water with chlorine, use of sanitary latrine, hand-washing before meal preparation, and hand-washing after defecation.

## DISCUSSION

The initial pilot of the CTI program in the Kathmandu Valley suggests that this type of cholera-control approach is feasible in an urban, developing country setting. Traditionally, divides between clinicians, laboratory staff, and government responders have existed in Nepal as they do in many other countries. Clinicians treat patients based on clinical symptoms and they are often discharged before laboratory results are available. Combined with little to no contact information collected on these cases at admission (address, telephone number, etc.), this scenario has made it very difficult to follow-up on cases in the past. This implementation of the CTI approach shows the potential to alleviate these issues, as participants at every level were required to communicate results quickly to remain within the established guidelines. Flow of information was evident from hospital admission as RRTs were able to interview 78% of patients before discharge, and this information was successfully translated into household-level, and in some areas, community-level responses. This program was also being implemented at a time when the health system was stressed post-earthquake, making the successes of the program even more promising.

Despite this being the first time such an approach has been used to control cholera in the country and the absence of a vaccine to fully implement the intervention, the program contributed to a heightened awareness of cholera and AWD in the Kathmandu Valley among government officials, hospital staff, and local NGOs. The creation of a cholera task force within the ministry’s enteric disease steering committee during the CTI project further engaged these key players and the designation of focal points at sentinel site hospitals empowered hospital staff to report cases daily. This increased awareness is further evidenced by the subsequent workshops held among these stakeholders to discuss the lessons learned from the CTI program and the resulting creation and endorsement of the country’s first national cholera control strategy following the 2016 cholera season. This increased awareness also played a large role in the ability of the RRTs to mount a comprehensive response, rather than compartmentalized responses at each level of the health system.

In addition to ensuring communication between historically disparate stakeholders, the evaluation of the CTI approach assists in the identification of bottlenecks for time-to-response. Diagnostic capacity is largely lacking at the hospital level, and although a few hospitals do have the capacity to perform culture confirmation of cholera, the time required to receive culture results diminishes the effectiveness of a response. Rapid response teams were able to respond to cases within an average of 4 days from hospital admission, but three of those days were typically spent waiting for a culture result. This highlights the need for an expansion of RDT at the hospital level for surveillance and response purposes, as well as the need for a reliable rapid test. The use of point-of-care RDTs would simplify all levels of the cholera surveillance and accelerate the response system by allowing the laboratory staff to provide clinicians with a rapid diagnosis, and the medical recorder with a diagnosis that warrants immediate report to the EDCD response team. An intervention could be implemented in the affected area within hours, as opposed to days, potentially preventing additional cases.

In many cases, daily reports came from the large government hospitals, but for other health care providers, timely reporting remains a major issue. Staff reported being overburdened at the Ministry of Health and in the health facilities, making it difficult to encourage hospital reporting and government follow-up when the reports were not presented. With an at-risk population of 18.5 million people, one potential solution could be to increase or re-route manpower to specifically work on AWD and cholera surveillance at the district level during the monsoon season to ensure all cases are being identified, reported, and responded to. Lessons could also be taken from hospitals that consistently reported daily even when no cases were seen to create trainings for the less-responsive hospitals.

The bar set for a “rapid” response under the CTI approach was to respond to the home within 2 days of a case presenting to the facility. Rapid response teams were able to perform thorough investigations at the homes of just over half of the confirmed cholera cases, but it took twice as long as planned. Several issues were at play here that can ultimately be traced back to the hospital-based surveillance. First, the vast majority of cases that could not be followed-up with household investigation were because of a lack of, or incorrect, contact information for the patient in the medical record. This is often a direct result of an overburdened and understaffed hospital where the accuracy and completeness of patient information is not a top priority. The EDCD officials’ uncertainty around the effectiveness of RDTs for case confirmation was a second time-limiting factor. Their preference to wait to initiate the household intervention until the case had been confirmed by culture led to major delays in response. To alleviate this hesitancy, culture was performed in parallel with the RDTs, resulting in a sensitivity and specificity of 90% and 70%, respectively. These results indicate that a system in which a response can be initiated by RDT result would lead to improved performance; culture can still be used at the national level as gold standard confirmation for official reporting. Especially in cases where an outbreak has already been detected and confirmed by culture, RDTs are a very efficient surveillance tool. Although false positives may lead to an increase in case load, these instances can be reduced by adding an enrichment step for 6 hours before testing. Last, it took as many as 6 days to respond after culture confirmation. There were only two central-level RRTs devoted to cholera response during the CTI implementation and as the outbreak progressed and daily case count increased, it was more difficult to keep up with household investigations. Manpower issues are a common constraint in Nepal, and one that will need serious commitment from the government to overcome.

At the time of this intervention, there was no straightforward mechanism for initiating rapid interventions in Nepal’s health system. Before implementing WASH interventions, planning meetings needed to be held at the district level, even when the same intervention had already been carried out in another area of the same district weeks earlier. On average, it took teams 9 days to agree on a location to perform an intervention and obtain the necessary approval to carry it out. Although ensuring a quality response is important, there is a need for standard interventions to be agreed on and planning meetings and trainings to be held before the cholera season. This recommendation was presented to Ministry of Health officials, and discussions on how to implement this change are ongoing.

The WASH program was designed to target those households immediately surrounding a case, however, interventions were planned and implemented more broadly. The intention was to reach more people at risk, but the result was low coverage of the intervention in the target population (neighbors of a case). In addition to needing a more rapid response, these results highlight the need to narrow the population target for the interventions. It is also notable that among those who recall receiving a WASH intervention, few cited receiving specific education on cholera, which shows a need for better training in the delivery of this important messaging. However, recall of the interventions themselves was associated with higher cholera-related knowledge.

A key element of the CTI response was the monitoring of water sources. Nearly all sources were contaminated beyond levels safe for human consumption; however, only three water sources were found to be positive for *V. cholerae* O1 during the household investigations. This sheds light on the state of the water system in Nepal, and the vulnerability of the nation’s poor. It is no surprise that improvements are needed in the water and sanitation infrastructure around the country, and steps are being taken, especially in light of these new data. Interventions such as WASH and OCV should be leveraged to prevent morbidity and mortality while those improvements are made.

### Limitations.

The inability to obtain OCV within the program period was a significant obstacle, but it led to a discussion of the need for a small national vaccine stockpile. Without adequate knowledge of disease burden on which to base a pre-emptive vaccination campaign, the proposed reactive strategy provides an efficient alternative. To overcome this, a small stockpile would allow the Ministry of Health to respond quickly to seasonal outbreaks, but would also provide a safety net in the event of a large outbreak while more resources are being requested and obtained. Using vaccine within this approach would also require the targeted response that was lacking in this implementation of the CTI WASH intervention. The approach was designed to target the neighbors of cases, those living within approximately 100 m of a case household. This distance was chosen by experts at the ministry of health based on their knowledge of the population density of the Kathmandu Valley and a goal of vaccinating approximately 1,000 people per ring. The logistics of targeting OCV to a small population, and how large that population should be, are complicated and will require further study. It is clear that the selection of ring size is both population and resource dependent, and will likely need to conform to administrative boundaries (such as a ward) for ease of implementation at scale.

In 2016, the CTI intervention was implemented as a program, not a research study, by the ministry of health, leaving many options open to interpretation by the implementers rather than being tightly controlled by study staff. Although this allowed for the feasibility assessment to be more true to life, it limits the ability to draw certain conclusions, especially in terms of the WASH response. Evidence surrounding effective WASH activities and implementation strategies is lacking.^17^ Although the results here show an association between recall of the intervention and cholera knowledge, a true assessment of the intervention will require a tightly controlled, randomized study design and is planned for the future.

Another potential limitation was the 6- to 8-month lag between the implementation of the WASH interventions and conducting the household survey. It is possible that individuals who recall the intervention are also more likely to recall the cholera-related knowledge, leading to inflation of the estimated ORs. Despite the implications for recall and therefore the reliability of the coverage estimate, the results of the knowledge portion of the survey are informative. Whether or not the family received or remembered the intervention itself, the results reveal the proportion of the population that has the knowledge necessary to protect themselves and their family from cholera. It has also been argued that surveys are inadequate for collecting data on the personal issues targeted here, such as hand-washing, food hygiene, and proper sanitation practices, because rates of such behaviors are often overestimated.^18^

Finally, timeliness and practicality were the main criteria used to determine feasibility of the CTI approach, however, cost is also a large determinant of the feasibility of any public health program that was not considered in this analysis.

## CONCLUSION

The CTI shows promise as a feasible strategy to unify effective cholera control procedures. We understood that this approach would represent a major change in the present procedures for cholera management, because case management, laboratory assays, and public health response are not generally tightly coordinated. The clinician would need to identify the case quickly and arrange for a rapid test to be carried out. The technician carrying out the test would notify the EDCD of the positive case, and the CTI RRT could quickly (within 2 days of the case coming for treatment) visit the neighborhood and implement within this neighborhood an integrated intervention package including WASH, health education, community mobilization, and vaccination. On its first implementation, this timeline has been extended, but through this evaluation we have revealed the current weaknesses in the cholera surveillance system and identified concrete areas for improvement seen in Table 5. These issues with the response were extensively discussed post-monsoon season and solutions were integrated into the country’s first national cholera control plan. Armed with this experience, increased awareness, available doses of vaccine, and a government and stakeholder-endorsed plan, the CTI approach has the potential to prevent the spread of cholera in the Kathmandu Valley, and eventually around the country.

**Table 5:** Key recommendations

1	Decentralization of case confirmation via culture to the provincial or district level
2	Expansion of rapid diagnostic testing at the hospital level for surveillance and response purposes
3	Focus on obtaining contact information at the hospital level for case follow-up
4	Re-routing manpower to cholera surveillance at the district level during monsoon season
5	Standardizing interventions and training implementers before the cholera season
6	Create a small national stockpile of cholera vaccine to aid the ministry in responding quickly to seasonal outbreaks
